# The Impact of Attempted Suicide on Young Adults: Learning from the Lived Experiences of UK Students in Further and Higher Education

**DOI:** 10.3390/healthcare13243222

**Published:** 2025-12-09

**Authors:** Jude Smit, Lisa Marzano, Erminia Colucci

**Affiliations:** Faculty of Science and Technology, Middlesex University, London NW4 4BT, UK; l.marzano@mdx.ac.uk (L.M.); e.colucci@mdx.ac.uk (E.C.)

**Keywords:** suicide, suicide attempt, student, education, Further Education, Higher Education, lived experience, mental health, stigma

## Abstract

**Background/Objectives**: A need for suicide prevention and postvention strategies in Higher Education was identified in the United Kingdom and has more recently been addressed with policies that provide national guidance for organisations. However, a paucity of qualitative research related to the lived and living experiences of attempted suicide in young adults remains. The experts in attempted suicide are those who have experienced it and the objective of our study was to learn from these lived experiences, with a particular focus on 16–25-year-olds in Further and Higher Education. **Methods**: The research presented in this article was part of a nationwide study in the UK which included 21 semi-structured interviews with young adults who met these criteria on the impact of attempting suicide on a personal, interpersonal, and institutional level, and support service experiences and engagement. It aimed to answer two key questions: 1. What can we learn from the lived experiences of young adults who have attempted suicide? and 2. How can these findings be applied to better meet the needs of young adults experiencing suicidal thoughts/behaviour in Further and Higher Education? **Results**: Reflexive Thematic Analysis was used to analyse the data, and four main themes were identified: firstly, the impact on ‘self’, including emotional and psychological impact; secondly, ‘others’, revealing the impact of and on relational factors, stigma, and judgement; thirdly, ‘systemic’, which highlighted support service experiences and barriers to accessing and engaging with possible support, and, fourthly, ‘what helps or could help’ on a relational, educational, and institutional level. **Conclusions**: The findings from this study generate new insights into this under-explored and stigmatised area and point to key barriers to support and gaps in service provision. Attempting suicide is one of the highest risk factors for a death by suicide and this study highlights the need for additional policy and support guidance for attempted suicide in Further and Higher Education.

## 1. Introduction and Context

Much has been written about suicide and suicidal behaviour in post-16 education [1,2,3], but with less focus on lived and living experiences, particularly of people who have attempted suicide. Although models and guidance for student suicide prevention within Further and Higher Education exist [4,5] and university guidance, such as ‘Suicide-Safer Universities’ [6] and ‘Responding to Suicide: Advice for Universities’ [7], has been created, engagement with and access to support continue to be the greatest barriers [8]. “Suicide is a whispered word” [9] and its taboo nature create internal and external barriers that silence it. Although the lived experiences of suicide are unique to the individual, the social determinants of suicide [10] need to considered.

Existing literature identifies the impact of stigma on interpersonal relationships and potential help-seeking as a key contributing factor [11,12], with transitions between organisations heightening loneliness and social isolation amongst new peers, staff and, structures [13]. Such loneliness is, in turn, a known risk factor and can increase the risk of suicide following an attempt [14,15]. For example, research has pointed to the association between social identity [16] and a lack of connectedness [17] or sense of belonging, including institutional belonging and suicide attempts, and highlighted the importance of social support [14].

Self-stigma and self-perceived social failure [14,18] can manifest as an individual’s internalising of externally perceived stigma and, consequently, an erosion of self-belief, leading to self-discrimination [12]. The prevalence of self-stigma also challenges self-compassion [19,20,21] and a lack of self-efficacy can be a factor in not seeking help. Low, or lack of, self-efficacy and the impact of trauma-related shame [18,22], bullying and victimisation [23] have been found to lead to increased emotional distress and add to feelings of hopelessness. The impact of pressures and expectation on academic functioning and performance are recognised, leading to increased anxiety and stress associated with perceived academic achievement [24,25,26].

Public and personal stigma [27] have also been linked to barriers to the use of mental health services within colleges and universities [27]. Some studies have found that young adults thinking about suicide are less likely to confide in mental health professionals [16,28] and that discrimination, stigma associated with mental illness, social identity, and negative social stereotypes [16,29] can lead to the fear of friends or family finding out [30]. This creates further barriers to potential help-seeking [16]. Interpersonal interactions that are perceived to be negative, critical, or rejecting are also found to increase the risk [21]. Students with disclosed or diagnosed mental health challenges are twice as likely to leave university without completing their degree [24].

Suicide remains often pathologised [31]. Defining suicide in this way is, however, reductive and reinforces prejudice, stigma, and self-stigma by not accounting for the contributory social, cultural, and political factors [32,33]. Suicidal thoughts, feelings, and behaviours can be experienced by anyone for a variety of reasons at any point in their life.

By focusing on lived and living experiences, we can gain unique insights into the impact on a person’s sense of self, sense of others, and ways of being in the world, as well as identifying what has helped someone to stay alive [34]. The aim of this study was, therefore, to explore what we can learn from the lived experiences of attempted suicide in young adults, with a particular focus on their experiences of, and engagement with, Further and Higher Education. Both lived and living experience are referred to in this article: the former refers to those for whom the experiences reported are in the past and the latter those living with suicidal thoughts, feelings, and behaviours in their present.

## 2. Methods

### 2.1. Design and Sample

As part of a wider UK-based mixed-methods study of young people’s experiences of attempting suicide whilst in Further or Highter Education [35], we conducted semi-structured interviews with 21 students with lived and living experiences of suicidality.

Participants ranged from 16 to 25 years old and included people with neurodivergence, diverse genders, ethnic backgrounds, and cultural beliefs. To protect anonymity, an aggregate of participant characteristics has been included as Table 1.

They were recruited via a national survey [35], which was promoted on social media and via organisations and individuals that supported the study (e.g., through relevant special interest groups and charities, such as MQ Mental Health Research). The survey was designed to gather experiences of attempted suicide amongst young adults at colleges and universities, and included an invitation to take part in a follow-up interview. In compliance with university ethics regulation, we discouraged participation from those who had made a suicide attempt within the previous 6 months. In 3 cases, participants who had made more recent attempts still wanted to be involved and asked if they could be contacted when 6 months had passed. This was honoured, as it felt important to be able to include everyone that wanted to participate. The interviews took place between January 2020 and May 2021. The study was non-selective, providing participants met the study criteria: aged between 16–25, in Further or Higher Education, and had attempted suicide. Everyone who came forward was included in the study.

### 2.2. Materials and Procedure

A semi-structured interview schedule (Supplementary File S2) was developed to create a framework and structure to the interviews. This included questions about how many suicide attempts they had made and how many of these they had disclosed to their college and/or university. They were also asked about their experiences of transition between school and college/college and university and any challenges experienced in college/university. Participants were also asked about support and suicide attempts, how suicide attempts impacted their college/university experiences and if support was available, what was helpful, what was unhelpful, or what could have been helpful. Questions included a focus on lived experiences and the impact on oneself, what participants would like others to know about attempted suicide, and any myths they would like to challenge or dispel.

Participant wellbeing was at the heart of the interviewing and wider research process. For example, participants were provided with extensive information in advance about the study, their voluntary involvement, and data use. To give time and space to hear what participants wanted to share, no time limit was set and interviews lasted between 40 min and 2 h. For some, this was the first time they had spoken about their experiences. Containment and safeguarding remained a priority. Time was given at the start to ask any questions and to debrief at the end of the interview, as well as consideration given to any potential need for ongoing psychological support. All interviews were conducted by JS, who is a psychotherapist/integrative arts psychotherapist and had access to clinical supervision. Consolidated criteria for reporting qualitative studies (COREQ) can be found in the online appendix (see Supplementary File S1) [36].

### 2.3. Ethics

This study was approved by the Middlesex University Psychology Research Ethics Committee (ref. 7390). All research materials and procedures were developed in consultation with a participatory research group (PRG), which included people with lived experiences, Samaritans, a mental health and education visual media correspondent, academic experts, and senior college/university welfare and safeguarding staff. Participants gave written informed consent, with the option to withdraw from the study within 8 weeks of participation (which no participant took up). Data protection and protecting identity were carefully considered. Any identifiable material was removed or redacted to ensure anonymity.

### 2.4. Data Analysis

The interviews generated a large amount of data. Reflexive Thematic Analysis [37] was used, drawing on a critical realist perspective [38,39]. This provides a robust and respectful approach to the complexity of lived experiences, taking account of the individual’s unique experience, whilst deepening our understanding of the underlying contributory causal factors. It enabled a reflexive process where semantic and latent coding [37] was possible and supported an inductive approach, looking for conscious and unconscious communication to capture both explicit and implicit meaning. Reflexive TA takes account of the researcher’s role in the process of analysis and examines how potential subjectivity could influence emergent patterns. Researcher bias was considered, especially as the lead researcher had a long working history with people experiencing trauma and suicidality. Keeping track of coding and ensuring consistency and rigour was paramount. Although frequency of codes is not a specific feature of reflexive TA per se, it was helpful to keep track of particular codes that recurred. To ensure robustness and maintain internal integrity of the analytic process, MAXQDA 2022 [40] software was used.

The structure of Reflexive Thematic Analysis (TA) was followed [37]: 1. familiarising yourself with the data, 2. generating initial codes, 3. generating initial themes, 4. reviewing themes, 5. defining and naming the themes, and 6. writing up. All interviews were transcribed and read and re-read as a whole, and any initial codes and overarching ideas and themes noted. Next, 10 interviews were selected across timeframes, age, level of study, gender, intersections, and length of interview. These were coded and recoded inductively in-depth. This generated a large number of codes which were then reviewed, and emergent themes identified. Codes were further refined to detect overlap or similarity. Where appropriate, codes were merged. The remainder of the data were then analysed and codes tracked, looking out for any new coding or possible themes. Finally, all data were reviewed again, and themes and sub-themes were reviewed, defined, and named.

Once this process was complete, data were further interrogated, drawing on Corbin and Strauss’s grounded theory coding paradigm [41]. This was adapted to provide additional structure, as a framework to help organise the themes and how to best present these to form a coherent narrative and draw most meaningful conclusions.

Saturation in qualitative research is a recently debated concept [42,43]; however, data were analysed and re-analysed until no new themes emerged. Data codes and themes were reviewed by the research supervisors (LM and EC) and a specialist MAXQDA coach.

## 3. Findings

**Figure 1:**
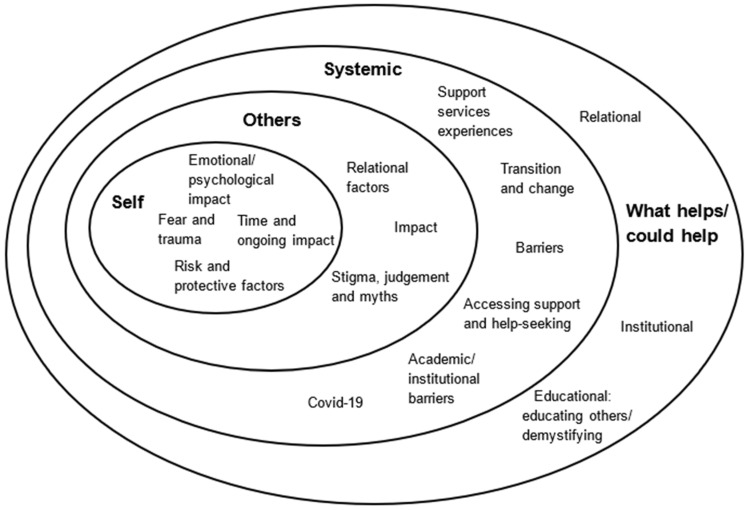
Impact of and on attempted suicide in Further and Higher Education students.

We identified four key overarching themes in the interview data: self; others/relational; systemic, including education, health, and barriers; and what helps or could help (Figure 1).

Participants’ accounts showed the emotional and psychological effects of attempted suicide, and factors contributing to it, on a person’s sense of self and ways of being. The diagram (Figure 1) shows how the overarching themes of self, others/relational, systemic, and insights into what helps or could help all intersect, with commonalities that shall be discussed in more detail.

We will first discuss the impact of and on Further and Higher Education, followed by further contributing personal and interpersonal factors.

### 3.1. Support Services Experiences and Barriers

Initially, the plan was to code support service experiences for each educational sector, but, as the participants were not evenly split across sectors, this was not possible. Consequently, systemic issues have been reviewed more broadly, but factors specific to a particular sector, such as experiences of transitions, were identified.

As the interviews progressed, it became clear that this broader overview of support revealed patterns that may not have emerged otherwise. Participants relayed negative and positive experiences within academic and medical systems and how both impacted each other, including barriers and a lack of support availability or provision. For many, previous experiences of systemic stigma and judgement originated much earlier, at school and in child and adolescent services. The impact and implicit fear of these early experiences and fear of negative consequences transferred to organisations later on and inhibited disclosure.

Barriers often pre-dated the time at college and/or university, with many negative school experiences shared. A participant reflected:

“*I don’t feel ashamed about it anymore because I think it’s a reflection of how I felt at the time and how I was being handled at the time by my school.*”

(R0015, female, H.E.)

Although the study was not related to school or school age, its recurrence across all interviews is relevant as these experiences became future academic and systemic barriers. Examples given were oversharing without consent, not being able to go to school, information not being responded to, and feeling marginalised, unmet, misunderstood, shamed, and not being believed about the severity of experience. These manifested in ongoing and future educational barriers and internalised self-stigma. A participant commented:

“*Just because someone hasn’t succeeded at completing suicide doesn’t mean that that wasn’t their goal, that someone wasn’t in a really dark place.*”

(R0020, female, H.E.)

Systemically, participants noted a lack of medical services, support availability and gaps in service provision across all sectors. This included a lack of immediate support, or joined-up, long-term support and parity between physical and mental health. They noted limited choice of support options and capacity, with long waiting lists. Participants described feeling deprioritised and stuck in a system, being moved around services or being referred on when considered too high-risk or felt to be “too serious” once they mentioned suicidal thoughts. Services and support were not easily accessible and what was offered was limited or cut off abruptly. Support was generalised and not ongoing or not targeted at suicidality:

“*It wasn’t very helpful because I think they were just sort of trying to do a one size fits all approach.*”

(R0016, female, H.E.)

Some participants had made numerous attempts at help-seeking, but none was available at the time. A participant recalled:

“*She said that with waiting lists and my severity that it was unlikely that it would be anytime soon, and that leaves you thinking… what other solution do I have and that is suicide, which is what I turn to.*”

(R0013, female, F.E.)

This eventually stopped another participant from reaching out, “*because at one point I’d had a mental health assessment nine times in a month and each time I got sent away, said go home, and that was me asking for help*.” (R0005, male, H.E.)

### 3.2. Transitional Barriers

Participants largely felt unprepared for change and transition and experienced anxiety, nervousness, and apprehension about change, and fear of not coping. Participants described particular difficulty at the start of college and/or university and noted an increase in academic demands across all transitions as well as a need to get used to academic demands over time. There was a lack of information about support ahead of the transition and subsequent support with the transition, and a feeling that more could have been done. One participant recalled, “*I arrived there and there was so much they could have done to support me*” (R0014, female, H.E.). Participants expressed a need to raise awareness about transitions, so that people can be more informed and mentally prepared. Another participant reflected on the implications of this:

“*I didn’t mentally prepare, and so I think that was definitely a factor because I didn’t give myself the time to accept that I will be overwhelmed and that I was scared about it, I just pushed that down.*”

(R0018, non-binary, F.E.)

Participants’ attempts at support-seeking were evident and raised issues around the accessibility of information and thresholds to services, knowing when to seek support, not meeting the threshold for mental health support, or a need for diagnosis to access support being presented as barriers. Not knowing what support was available or who to contact led to feelings of disillusionment and defeat.

Support in academic establishments was described as having a limited focus on mental health support, with educational support services advertised as disability support, not mental health support. A lack of signposting, information sharing, and inaccessible language were often experienced as barriers and equality issues were raised, as expressed by this participant:

“*They need to be signposting services, they need to be making sure that there are services there.*”

(R0014, female, F.E.)

Some highlighted the social challenges with transitions and expressed feeling lonely and needing social contact, adjusting to living with new people, not able being to settle, and the ongoing challenge of being in university: “*university is hard*.”

Participants identified positive experiences that broke down some of these barriers. They appreciated supportive tutors and teacher sensitivity, a collaborative approach, with targeted academic support and the proactive sharing of resources and helping to manage expectations.

### 3.3. Relational Barriers

Overarchingly, relational aspects appeared to affect participants the most and challenges included feelings of obligation, peer-pressure, people-pleasing, and sacrificing the self. Power dynamics and negative responses from others when help-seeking at points of crisis were at times described as catastrophic. Needing to justify oneself and “*not being understood and not being… taken seriously*” created barriers (e.g., “*I won’t go to my mental health team or my crisis team now because…they tell me that it’s not a crisis and if I was serious, I would just go and do something, I won’t go to them*” (R0007, female, H.E.). This also applied to going to hospital at times of crisis.

Relational factors presented as barriers, both academically and institutionally. Participants described feeling shut down by staff and expressed mixed experiences of educational responses post-attempt. They recalled the need for justification to obtain university support, intrusive questioning, and having to share information for university admin purposes and the challenging process to obtain academic extenuation; as indicated by one participant, it felt like “*an interrogation almost like why do you need the extension*” (R0011, female, H.E.).

The importance of the relationship and relational elements affected support. Building trust takes time and support needed to come from someone you know or can get to know. To keep having to tell one’s story due to a lack of consistent support created barriers and reinforced attachment challenges. A participant commented, “*even though there was other support, I kind of needed someone which I had a really good relationship with, and I didn’t feel I had that specifically*” (R0010, female, H.E.). The inconsistency and changeability of support and the person who was seen reinforced feelings of abandonment and fear that support be withdrawn or a support person would leave.

Some participants reflected that disclosures were not always followed up, or they were not checked up on after they had shared that they were experiencing difficulties/challenges. Others described going back after an attempt and staff being ill-equipped to navigate these conversations, resulting in restrictions on courses and suggestions to leave the course; for example, “*with tutors trying to get me to drop out, I feel like I’d have to become like this perfect student…I feel like I’m not allowed to struggle*” (R0012, female, H.E.). Additionally, disclosures were reported to impact university accommodation and, for some, resulted in being asked to leave. A participant explained:

“*It’s basically just the fact after one mention of suicide they decided to chuck me out and they wanted me off campus as well, the university wanted me out of the halls of residence because I was too risky.*”

(R0005, male, H.E.)

Barriers to accessing support were closely linked to barriers to sharing and help-seeking. Disclosure felt complex, with difficulty speaking about the attempt and fear of consequences from opening up to student services. Some expressed unease at not knowing what had been shared about them with an educational establishment. Participants reflected on fears of parents or other people finding out, or the consequences of telling, such as choice being removed. Participants felt disempowered when people were told about their attempt(s) without their consent, or if they were forced to tell or disclose because of circumstance.

Expectations from support providers often added pressure to share more than participants felt comfortable. A power imbalance presented itself and participants felt pressured to talk about attempts, describing this as intrusive and feeling under surveillance. Some conveyed the negative impact of others taking control and gave examples of parents being told about their attempts without their choice or consent. A participant reflected:

“*I remember being careful about what I said in terms of suicide because I didn’t want there to be consequences.*”

(R0015, female, H.E.)

Detachment arose from a lack of communication and connection to people and to the wider world, such as a lack of family or home support and community. Participants expressed a want and need for help and de-escalation; they were willing to ask for support but, in many cases, found the barriers too challenging. They identified a lack of respect for suicidal people, space to talk, and validation, as well as preventative measures post-attempt and support or specific support service for survivors of suicide attempts.

Missed opportunities for intervention came up repeatedly and were the most challenging to see emerging across the data. Although this varied from person to person, it was clear that there was an evident and apparent timeframe where intervention could have been possible. A participant observed, “*I was actually warning people six months before it happened…saying I’m going to crash, this is going to end badly, I’m going to crash!*” to both their school and a psychologist (R0008, transgender male, F.E.).

Some participants were aware of college/university support, but they were not accessing or engaging with services and described difficulty communicating. The immediacy of support availability was evident and there was a need for earlier intervention that was targeted and accessible. For some, this meant in-person rather than phone support, and, for others, text-based support. They described a need for agency, empowerment, and independence and reinforced the need to be seen as an individual and to be mindful of different experiences. They wished to take their own initiatives and have a choice in telling people about their attempt, as well as a say in support options and action planning.

Some participants described positive experiences of reaching out for support and a change in perspective, feeling more connected to support post-attempt. Service and organisational approaches deemed helpful offered proactive, solution-focused, directed support, where participants felt prioritised, respected, and validated with dignity. A participant explained this:

“*Targeting the problem from many different aspects, rather than just kind of what just happened and how do we get over that.*”

(R0011, female, H.E.)

It took a long time to find the ‘right’ support and interviewees said that what was most helpful was regular, ongoing support, particularly post-attempt. Again, human, interpersonal qualities were most important, such as acknowledgment, understanding, and being cared about. Participants expressed a need for trust, compassion, kindness, and respect, including respect for personal space. Staying out of hospital was a motivator.

When considering the relational impact, what particularly stood out was the positive impact of one specific person alongside them at the time, for some, still remembered years later. The human, relational qualities were most impactful. A participant remembered someone’s kindness, care, and compassion:

“*It’s not her role to support students in that way but she did, and I found that more helpful than any other support I got.*”

(R0011, female, H.E.)

Additionally, the difficulty speaking about the attempt or attempts to others, but the willingness and openness to share in the interviews themselves were notable. Some spoke about their lived experiences for the first time. Participants fed back positively about being able to speak freely and openly in the interview and to share as much or as little as they chose to, without pressure or expectation:

“*It doesn’t feel as uncomfortable because I know I’m just answering a question, we don’t have to go into depth about it…it’s very much like I’m just explaining it as it is and so it feels a bit more, I guess, controlled.*”

(R0018, non-binary, F.E.)

### 3.4. Stigma/Judgement/Myths

Participants were asked to comment on any myths or perceptions they wished to challenge. Negative assumptions and negative reinforcement were prevalent and early experiences starting at school had developed into withdrawal, self-stigma, and isolation. Participants remarked on the negative impact of biased media representation and public perception, people’s judgement, and misunderstanding about suicidal behaviour as a way of communicating distress, with comments such as “*attention seeking*”, “*cry for help*”, and “*not being serious*” about the attempt if they survived it. One participant clarified:

“*If you think to yourself, oh that person just wants attention, oh my God, how bad is that, that that person is hurting so much that the only way they felt like they could reach out for help was putting their own life at risk…we all want attention.*”

(R0010, female, H.E.)

The impact of misconception was discussed and words such as “*selfish*” and “*weak*” were challenged.

The taboo nature of suicidality and fear of judgement and stigma led to hiding or having minimised the attempt. Participants professed that many people experience suicidal thoughts, feelings, and behaviours, but few speak about it. Stigma that derives from myths, misinformation, misconception, and judgement leads to self-stigma and internalised shame that silences. A participant summarised:

“*I think I had a lot of shame to do with suicide and self-harm, just from the way that people had responded to it.*”

(R0015, female, H.E.)

Stigma and judgement can make an attempted suicide a dehumanising experience at a time when someone is already at their lowest point and contribute to further feelings of hopelessness and defeat. Participants emphasised social injustice and being reduced to statistics, reinforcing the dehumanising impact of post-attempt experiences. They also noted that a risk-averse narrative and unhelpful platitudes were alienating, stating “*I think the stigma only harms*”. Unconscious bias and stigma included stereotypes about increased suicide in people with protected characteristics.

Participants described feeling ‘different’ to others and not fitting in. They cited negative experiences of the social environment and spoke of global inequality. What is clear from the data analysis is the major impact a person’s response to someone who has attempted suicide can have. One participant summarised:

“*For me it’s never really been the attempt itself, it’s been the reaction from other people that’s been the biggest deal out of any of it.*”

(R0008, transgender male, F.E.)

Assumption and myths were seen as presenting significant barriers to speaking about suicide. In the words of an interviewee:

“*Once society has changed their view on suicide, and then more people will come out and it doesn’t have to be like…even me…I’m too scared of their reaction. It shouldn’t be that way at all.*”

(R0005, male, H.E.)

A lack of understanding and awareness was expressed and attributed to inexperience within medical and mental health teams and insufficient training for medical staff or tutors. Negative assumptions/comments and advice reinforced feeling of shame. One participant explained:

“*I still feel quite a lot of shame about it, like I don’t tell people when I get to that level of low.*”

(R0015, female, H.E.)

It was difficult to hear how isolated and alone participants had felt and the barriers that stigma and judgement created, making it feel impossible to reach out: “*people that are considering suicide do not have the capacity to reach out and get help*” (R0013, female, H.E.). At this point, “*they need people to reach out to them*” and, in some cases, it took an attempt to realise that people cared.

In this context, suicide prevention campaigns were deemed largely ineffective. Many were said to be focused on encouraging people to reach out for help. Most participants voiced that, particularly, in challenging times, this could be the hardest thing to do:

“*One of the biggest things for me, and I know for a lot of other people who go through things like this, is reaching out is the hardest part.*”

(R0015, female, H.E.)

The need to demedicalise suicide was clear, with misconceptions about who can become suicidal questioned: “*it can happen to anyone*” (R0013, female, H.E.). Participants made the distinction between thoughts, feelings, and actions and they challenged the assumption that having future plans in life is an indicator that someone is not actively suicidal.

### 3.5. Fear and Trauma

Fear of stigma and judgement was a significant barrier to speaking about challenges faced and accessing support, both at the time of and after their attempt. Participants described feeling threatened, fear of failure, fear of getting worse, and getting things wrong. Examples of abuse and the impact of early childhood experiences affected the person’s sense of self/other/world, including unresolved trauma and not being believed about traumatic events. Some identified a specific event shortly before an attempt and also post-attempt trauma triggers, such as the sound of sirens, evidencing both the impact of traumatic events and the trauma and circumstances of the attempt itself. These included mixed responses to and from ambulance, police, and hospital staff interventions, and affected a person’s way of seeing oneself and others and future help-seeking.

### 3.6. Emotional/Psychological Impact

Participants discussed the impact of their attempt(s) on their sense of self, and ways of being and relating to others, society, and the world. This included the challenge of being oneself and needing to put on a mask. As one participant explained:

“*With my friends I was always the jokey guy…when I was at home, it was almost like flipping a coin…just you know the same coin different face.*”

(R0021, male, F.E.)

Covering up and masking led to a presented self [44], a silent struggle, where the extent of despair was underplayed. Another participant noted “*I kind of tried to smile my sorrows away*”.

The internalised message to ‘*stay strong*’ and having to keep going whilst experiencing despair affected functioning and enhanced feelings of perfectionism. This split fuelled a negative cycle of trying harder and further masking that increased hopelessness. As one participant expressed, “*it just kind of went into more kind of hopelessness*” (R0020, female, HE). This resulted in a greater need to withdraw, hide, and isolate from others. Participants identified shame, self-blame, disappointment in themselves, and having to live, or not living up to, expectations. An example of the impact was shared:

“*Often I know at least personally I wouldn’t want to speak to someone about that because I would feel probably guilty or ashamed.*”

(R0020, female, H.E.)

Others expressed guilt, grief, frustration, and anger, and/or described feeling selfish and unworthy of support, like a burden, like they were no longer needed, and like they had a negative impact on the world, or that they did not belong:

“*I think I’ve always felt like I was a mistake in the world or like an anomaly.*”

(R0018, non-binary, F.E.)

Participants felt misunderstood and described the impact on their self-worth and self-value, a loss of identity, and loss of themselves. Some felt defined by their attempt(s) and that suicidality was a part of themselves:

“*I think a lot of people kind of hold onto that pain and really internalise and fear letting it go, as if they’ll be letting go an important part of themself, and you know that pain is something that if people have gone through then it’s traumatic.*”

(R0016, non-binary, F.E.)

The unspoken and internalised invisibility of hurt, emotional weight, and pain was clear in many accounts. For some, this manifested in feelings of resignation and defeat. Functioning day to day whilst masking was hard and they described low mood and loss of interest, feeling lonely, isolated. A participant conveyed the sense of inevitability expressed by many and being at a ‘dead end’: “*I was so sad, and I was so empty*” (R0018, non-binary, F.E.). The extent of the despair affected self-care and self-compassion. They felt disempowered, lacking agency, rejected, and let down. Feelings of abandonment and ongoing hardship eventually led to disconnection, and detachment from self, others, and the world. Another participant described the tiredness of life [45] and stated, “*I was just kind of done with doing the same thing every day*” (R0011, female, H.E.).

The preoccupation and persistence in suicidal thoughts overwhelmed and participants felt tired, trapped, “*spiralling down*”, with no escape and “*not being able to see into the future*”. The extent of hopelessness and helplessness was clear and there was a need for a way out. One participant used a powerful metaphor to illustrate this point:

“*Say they’re trapped in a box and they’re kind of saying, OK, well you know suicide’s this trap door.*”

(R0020, female, H.E.)

Some participants stated that they had wanted to die, but others felt relief when they realised that they had not died. Participants discussed not wanting to act on thoughts. Suicide is “*not always wanting to die*” (R0009, female, F.E.), but a response to total despair and hopelessness. Some expressed needing to hit rock bottom before being able to find a way forward. They described not feeling able to be alone or to go to sleep, unable to trust or keep themselves safe (“*It’s really, really difficult to pause between that feeling and that action*” R0018, non-binary, F.E.), but, at the same time, unable to reach out and needing to fight and hold onto some hope that support was available.

Many participants described numbness at the time of attempting (“*It’s like I felt nothing, I was just completely empty. And I think that’s almost always the same feeling*” (R0018, non-binary, F.E.)), or once they realised that they had lived beyond the attempt:

“*I think I just didn’t really feel anything…it wasn’t like a sense of relief, it wasn’t a sense of regret, it was just absolutely nothing.*”

(R0016, non-binary, H.E.)

Some participants recalled feeling that a failed attempt meant that suicide was no longer an option (“*It’s the last option, so what do you do when the last option’s taken away, you literally feel like you have nothing left*” R0010, female, H.E.). This, in turn, reinforced negative self-belief, with punitive statements such as “*I can’t even get that right*,” whereas, for others, the lasting effect of a failed attempt was a driver for future attempts, for example:

“*My first attempt was like unfinished business, almost like a ghost I would say, like oh why didn’t you do this, you should have done it, you’re not good enough and so on.*”

(R0021, male, F.E.)

Many participants described feeling changed after the attempt and difficulty adjusting to life post-attempt, like a different person. In contrast to feeling defined by the suicide attempt, some participants described it with ambivalence as “*just something that happened…an event*.”

### 3.7. Ongoing Impact

Codes related to time, transience, and the long-term, lasting effects post-attempt highlighted the ongoing impact and complexity of suicide. Participants explained that the feelings are changeable and can fade over time, but a fear of the feelings returning remains. Participants were clear that an attempt is not an isolated event and that there are multiple contributing factors to suicide. A participant reflected:

“*I never expected it to get that bad I always believed I’d get through it somehow, because you know I’ve felt suicidal multiple times in my life and I’ve managed to dig myself out of that big hole and keep going.*”

(R0019, female, H.E.)

Nuanced differences in experience were identified, such as fluctuations in the intensity of suicidal feelings, levels of intent, and capacity to be proactive. These were largely impacted by relational factors.

Suicide was described in several ways, as a coping mechanism over time, as a way out/escape, as an impulsive act, or as a carefully planned process. As one participant explained:

“*My attempts aren’t necessarily impulsive, they tend to have months of build-up and then they kind of happen…kind of opportunistically but very planned…it’s not like I have an argument and I attempt.*”

(R0008, transgender male, F.E.)

Sometimes, life felt like an ongoing battle and the attempt was the result of utter hopelessness. Some were unable to identify a specific trigger and expressed that, in that moment, suicide was not rational, as they were not able to think. Others described it as a carefully thought-out process:

“*It was the weighing up of pros and cons, which for me felt very rational, rather than the emotional kind of feelings you associate with suicide.*”

(R0013, female, F.E.)

Other participants described living alongside suicidal thoughts, always there in some form as a part of someone’s life:

“*It’s so weird how you just like move on from that. But I think a part of me never really does. And I think there is a void from each time, and I do try to fill it with things…but it doesn’t work.*”

(R0018, non-binary, F.E.)

Adjusting to life post-attempt included conflicted feelings of regret, wishing it had worked, or a wish to attempt again. Some experienced this as an enduring, ongoing challenge:

“*A lot of the time people get through a suicide attempt and they haven’t had this epiphany, they feel the same, and being told by everyone they should feel grateful when it’s like, honestly I’ve never felt less grateful…It’s the last option, so what do you do when the last option’s taken away, you literally feel like you have nothing left.*”

(R0010, female, H.E.)

The length of one’s recovery time was a common theme and the challenge of needing to plan life post-attempt was clear. The month directly after and up to six months following an attempt were described as the hardest:

“*It’s not just the crisis point that’s difficult, it’s kind of the months after it that are most difficulty, at least in my experience.*”

(R0011, female, H.E.)

Not having expected to live beyond a death date compounded uncertainty about the future and was fuelled by ‘what ifs’ and ‘what comes next’. The unique nature of lived experience was noted, and the impact of living beyond a death date. The participant explained:

“*It felt like I had to plan my life over again, because in my head I wasn’t going to be here after that set date…it’s almost like you’ve got a life that I didn’t expect to have.*”

(R0011, female, H.E.)

Some participants spoke of the complexities related to not knowing, or having, a place in the world, or not having found one’s purpose yet. The data reflected endurance and patience, waiting for the future to take shape.

Changes in feelings post-attempt were identified. For some participants, an attempt was a turning point. They shared that time was helpful and made a positive difference, providing a capacity for reflection. They noticed a change in perspective and perception about staying in the world, focusing on life ahead, or life moving on or moving forward. In the words of one participant:

“*I’m glad that I got help now and that I survived it, because even though my life is not perfect, far from it…I’m trying to do a lot more with my life now…I wouldn’t have got where I was now.*”

(R0012, female, H.E.)

### 3.8. What Helps/Could Help

Participants were asked to identify what was helpful at the time and for any suggestions as to what could be, or could have been, helpful based on their past experiences. A participant summarised what many expressed, that, with more support, most of the attempts could have been preventable:

“*I feel like a lot of this could have been preventative had it have been from like the earliest start as possible.*”

(R0006, female, H.E.)

The need for support, particularly for ongoing support post-attempt, was evident. Consistency and a proactive approach, being reached out to, and the importance and impact of a specific person were especially noteworthy. The importance of systemic support and student services were recognised, both academic as well as pastoral support. Meeting individual needs, accessibility, and easy access to resources were key:

“*It would save so much time and effort and money and service space if they were more proactive from the beginning…they need to be engaging robust support, they need to be signposting services, they need to be making sure that there is services there, and that there are reasonable waiting times.*”

(R0014, female, F.E.)

Participants understood the pressure on services but stressed the need for “*quicker referrals*”, quicker response times, and proactively being reached out to. Transparency from the start and highlighting which services were available would help with this.

Preparing students in advance with transition support across organisations could be beneficial; a participant recalled that “*I think just being more educated on things is always the way forwards*” (R0017, male, H.E.). They needed to see and get to know the person within the institution offering support.

Again, the taboo nature of suicide was raised, and the need for safer spaces and a support network. A wish to be reached out to and for people to understand, for someone to be alongside them who was respectful, validating and reassuring, open-minded, and non-judgemental, and who could actively advocate for and engage in joining up support.

Human connection, interaction, and the physical presence of others fostered a sense of belonging and community. The availability of support was vital. Ideally, this would come from someone known, such as friends, partners, and parents, or professionals who were consistent and whose support was ongoing. Someone proactively asking about the attempt and looking beyond the surface presentation encouraged disclosure, openness about the experience, and reduced suicidality. The value of listening, and feeling seen and heard were stressed, as described by one participant:

“*Listen to people, rather than going off your own assumptions of what’s the right thing to do, ask them…listen to them I think is the most important thing to do.*”

(R0015, female, H.E.)

Addressing many of the issues raised earlier in schools would mitigate some of the challenges experienced later.

Joined-up support, with a relational, collaborative, flexible, co-created, holistic approach felt empowering and supported a sense of agency. Taking account of individual needs where organisations could put in place what students themselves considered most helpful, to “*tailor it to the individual…you can’t have dignity without person-centred care*.” (R0010, female, H.E.). With consent, carefully considered cross-college/university sharing of information with staff could be worthwhile. This would avoid the potential challenges in communication for both staff and the student, especially around administrative matters such as absences and late submissions. Having a GP on the campus was found to be helpful.

A wish for more specific, targeted support for survivors of attempted suicide was clearly communicated:

“*There is nothing for survivors afterwards, after the attempt, or to prevent that thought process from ongoing to going from between I feel like a coward to I need to try that again, which really makes me angry.*”

(R0006, female, H.E.)

### 3.9. Educating Others and Demystifying

Current education and awareness around suicide prevention were perceived as positivist, tokenistic, and not integrated. Participants stressed the importance of knowledge about mental health and the need for more trained professionals within institutions (“*although her compassion you know was there, she probably could have done with some training*” (R0006, female, H.E.)) Specific education and practical training about self-harm and suicide within all sectors was raised. Participants felt that training for tutors should be a pre-requisite. Additionally, they identified a specific training need for police and the availability of support and training for parents.

Some participants conveyed a wish to be involved in the development of training, raising awareness, advocacy, and activism, with the aim to challenge assumptions, destigmatise, and demystify, stating that “*I want to use my lived experience to help other people*” (R0012, female, H.E.). They believed that including people with lived experience in education, policy, and training would help others gain a better understanding and, thereby, feel more equipped to support when needed.

## 4. Discussion

The aim of this research was to present the voice of lived and living experience as accurately and respectfully as possible and to start building bridges towards a kinder, more inclusive approach to suicidality, breaking down barriers and opening channels for further discussion to take place to support colleagues and institutions with an immensely challenging task, faced with cuts to funding and service provision in all public sectors.

It has been theorised that, although risk factors for suicidality are experienced at an individual level, they are heavily influenced by social determinants [10]. As evidenced by the findings, our study provides new insights into the far-reaching impact of suicide attempt(s) on a person’s sense of self, others, and ways of being in the world, as well as one’s relationship with mortality, the future, and an unexpected life ahead. To counter this, we need to start breaking down barriers by viewing suicide more as a transitory state of being and acknowledging that some people live alongside suicidal thoughts, always there in some form as part of one’s life.

Literature suggests that the majority of students who consider suicide do not access mental health services [46,47] and that, in fact, those perceived to be most in need of services are least likely to access them [48]. Our study reiterates that past experiences of services and reaching out for support have been found to influence potential help-seeking and a positive experience was more likely to lead someone to engage with this [16]. Another barrier identified in the literature and supported by our study was not knowing about resources or the availability of college/university support services [49], as well as the lack of specifically trained and/or experienced mental health professionals on site [50]. Our study suggests that this remains the case and that there is a definite need for a change in the culture to proactive rather than reactive support strategies and reaching out to people experiencing suicidality.

Literature also suggests that students fear stigma from the university structures, with some evidence suggesting students feared that information sharing could affect academic progression and their sense of belonging [16,51]. Again, our findings confirm that this poses an ongoing barrier to disclosure and accessing support.

It has been suggested that, in light of the additional pressures of transitions between education sectors, a possible focus on early intervention for first-year students, and students identifying with one or more of the protected characteristics or mental health challenges [49] could be a proactive measure. Our study shows that the greatest need is to first remove the barriers to access and engagement with support. It supports the literature that draws attention to the need for an inclusive approach to interventions, rather than a generalised approach to suicide prevention, by targeting and tailoring interventions to meet the diverse needs and experiences of students, including students with any of the protected characteristics [52], and cultivating a culture of belonging and inclusivity.

However, as revealed by the findings, some of the support needs are specific to post-suicide attempts and thereby differ from prevention strategies and require a different kind of intervention than that outlined in existing policies [4,5,6,7]. This is particularly relevant when considering the challenges identified with the transition to university.

Furthermore, the adjustment to life post-attempt highlights the urgent need to be seen sooner and for consistency in support, and to take into account the recovery time and also the impact of trauma of the attempt itself. This will firstly need to start with destigmatisation, to break down some of the identified barriers and to encourage disclosure. Secondly, finding ways to promote a culture of ‘reaching out to’. And, thirdly, consistent, targeted, and focused support for attempt survivors that considers transitional support and ways for support to be joined-up, so that this can be proactively approached and address some of the mitigating factors such as social isolation, pressure, and destabilisation that inevitably comes with change. As evidenced by the findings, the relational qualities of people in support roles are central to this and how organisations can work towards removing some of the barriers that derive from stigma present internally and externally.

### 4.1. Strengths and Limitations

Within qualitative research, there is a tension to create translatable codes and themes, whilst still doing justice to the unique nature of lived and living experience. The relational approach to interviewing itself was a strength and supported the aim not to speak for, but to best represent the voices that shared their lived and living experiences with integrity and respect.

Although recruitment reached far and wide across the four nations of the UK, the authors acknowledge the limitations of the non-homogenous nature of the study and that we only heard the voices of people who came forward. Consequently, the findings presented are therefore not necessarily representative of all people experiencing attempted suicide in Further and Higher Education.

Not having an even spread across Further and Higher Education meant that sub-themes, such as transitions, could not be compared. Additionally, not clearly delineating questions about health and educational support meant that these could not be written about separately, unless a particular sector was explicitly stated. However, it is likely that the depth of feeling and wider context would not have been as clear if these questions would have been more structured.

As this was a UK-based study that refers to UK guidance and procedures, and a UK educational and mental health service context, this may make it more difficult for people in other countries to relate to some of the findings. However, many of the themes remain relatable.

### 4.2. Implications and Future Directions

The study was specific to ages 16–25, but participants were asked when they made their first attempt. Most participants’ first suicide attempts pre-dated their 16th birthday, with the earliest recorded at 12 years old. Experiences before F.E. and H.E. were noted and challenges at school and in child and adolescent services had created anticipation and apprehension that particularly affected help-seeking and engagement. Participants spoke of challenges at the time that had deeply affected them. The need for earlier intervention is clear and additional study into school-age suicidality would be beneficial.

Our study identifies a gap in guidance and policy for attempted suicide at university. This is particularly noteworthy, given that attempting suicide is a strong risk factor for a death by suicide [53].

Universities UK and their colleagues have done much work to create pre- and postvention guidance and policy. This has gone some way to address the issues raised; however, the pressure on support providers across all sectors is immense [54]. These operate from a reactive stance due to long waiting lists and financial constraints. A review of funding and human resource allocations is much needed.

Further training to educate about and destigmatise suicide is needed, so that staff can feel empowered to foster more inclusive, compassionate approaches [3,55]. The effects and impact of the depth of emotional pain and trauma on staff are not to be underestimated and it is encouraging that, since the start of the study, colleagues have focused specifically on this [56].

The identification that more targeted and specific support is needed for suicide attempt survivors highlights an area for further studies into the difference in experiences between suicidal thoughts, feelings, and behaviours and how best to support and meet these differing needs.

In a world that is increasingly looking to utilise AI, the human and relational aspects across all data cannot be ignored, particularly when it is so hard for people to reach out. To create a practice of reaching out, we need to reframe current narratives. Campaigns to ‘Ask Twice’ [57] are helping to encourage this on an interpersonal level, but this needs embedding on an institutional level. A recent announcement stated that a Multi-Modal Approach to Preventing Suicide in Schools (MAPSS) will be taught in schools, specifically targeted at Year 10 students, aged 14 or 15 [58], with trials planned until January 2026. Additional training in suicide prevention will be offered to teachers and parents. This feels to be a helpful step towards affecting change, but we will be interested in the feedback from young people, teachers, and parents with lived and living experience of/supporting individuals with suicidality.

Our findings suggest that the biggest challenge remains stigma and what is evident across the data is the need to depathologise suicide and address the fear that creates barriers to both support provision and the access to it.

## 5. Conclusions

Much of the literature and current policy frameworks focus on suicide prevention and postvention. Close analysis of these documents and the findings from our study highlights a gap in policy for students who have attempted suicide.

Efforts are already being made to fight the tide and the confirmation that one specific person can make the difference between life and death and can provide a glimmer of hope in a hopeless place. To honour the voices presented in this article, a participant quotation is included to stress this point:

“*So the main message that I would give to people who attempted suicide…you will meet hopefully that one person who will change everything in your life.*”

(R0021, male, F.E.)

Kindness and compassion, breaking down barriers, and challenging stigma all impact how a person feels met and understood in the world. It is hoped that this research supports a growing understanding in both education and society that the relational aspects are the biggest barrier, and that this article in some way goes towards bridging this.

## Figures and Tables

**Table 1:** Participant characteristics.

Area of the UK	Ages	Gender	Further/Higher Education	Ethnicity
London: 3 Southeast: 9 Southwest: 1 East Midlands: 2 Yorkshire and the Humber: 1 Scotland: 4 Northern Ireland: 1	16–18: 6 19–21: 4 21–23: 6 23–25: 5 *(N.B. 1 person included turned 26 by the time of interview.)*	Female: 13 Male: 3 Non-binary: 3 Transgender Male: 2	FE: 6 HE: 15	White British: 16 Any other white background: 2 Any other Asian background: 1 East Slavic: 1 Prefer not to answer: 1

## Data Availability

The raw data are not publicly available as they include qualitative quotes that could compromise the privacy of the research participants.

## Supplementary Materials

The following supporting information can be downloaded at: https://www.mdpi.com/article/10.3390/healthcare13243222/s1, File S1: Consolidated criteria for reporting qualitative studies (COREQ): 32-item checklist. File S2: Interview questions–Learning from Further and Higher Education students’ lived experiences of attempted suicide.
