# Supplementary material for: The Impact of Attempted Suicide on Young Adults: Learning from the Lived Experiences of UK Students in Further and Higher Education
Source: Healthcare (Basel). 2025 Dec 9;13(24):3222. doi: 10.3390/healthcare13243222 (PMC12733092; doi:10.3390/healthcare13243222)
Supplement: Supplementary file 1 [file healthcare-13-03222-s001.zip › healthcare-3941052-File S1.docx]

**Consolidated criteria for reporting qualitative studies (COREQ): 32-item checklist**

Developed from:

Tong A, Sainsbury P, Craig J. Consolidated criteria for reporting qualitative research (COREQ): a 32-item checklist for interviews and focus groups. *International Journal for Quality in Health Care*. 2007. Volume 19, Number 6: pp. 349 – 357

| **No. Item** | **Guide questions/description** | **Reported on Page #** |
| --- | --- | --- |
| **Domain 1: Research team and reﬂexivity** |  |  |
| *Personal Characteristics* |  |  |
| 1. Interviewer/facilitator | Which author/s conducted the interview or focus group? | See Manuscript |
| 2. Credentials | What were the researcher’s credentials? E.g. PhD, MD | See Manuscript |
| 3. Occupation | What was their occupation at the time of the study?  *Psychotherapist, Integrative Arts Psychotherapist, Senior Lecturer, Mental Health Advisor* | See Manuscript |
| 4. Gender | Was the researcher male or female? | Female |
| 5. Experience and training | What experience or training did the researcher have? | See Manuscript |
| *Relationship with participants* |  |  |
| 6. Relationship established | Was a relationship established prior to study commencement?  *The interviewer had written correspondence with interviewees in advance of interviews, introduced herself prior to the interview and discussed the research with the participants.* | Yes |
| 7. Participant knowledge of the interviewer | What did the participants know about the researcher? e.g. personal goals, reasons for doing the research  *Participants were told of the interviewer’s professional background and experience, reasons for conducting the research, and funding source.* | See answer in column 2. |
| 8. Interviewer characteristics | What characteristics were reported about the interviewer/facilitator? e.g. Bias, assumptions, reasons and interests in the research topic  *JS is also a Mental Health Advisor in Further and Higher Education and has 20 years of experience working with young adults experiencing suicidality.* | See column 2 |
| **Domain 2: study design** |  |  |
| *Theoretical framework* |  |  |
| 9. Methodological orientation and Theory | What methodological orientation was stated to underpin the study? e.g. grounded theory, discourse analysis, ethnography, phenomenology, content analysis | See manuscript |
| *Participant selection* |  |  |
| 10. Sampling | How were participants selected? e.g. purposive, convenience, consecutive, snowball | See manuscript |
| 11. Method of approach | How were participants approached? e.g. face-to-face, telephone, mail, email | See manuscript |
| 12. Sample size | How many participants were in the study? | See manuscript |
| 13. Non-participation | How many people refused to participate or dropped out? Reasons?  *Refusal to participate: n/a (given recruitment method). All participants wishing to be involved were included. No participant dropped out.* | See column 2 |
| *Setting* |  |  |
| 14. Setting of data collection | Where was the data collected? e.g. home, clinic, workplace  *University premises, Samaritans premises and online.* | See column 2 |
| 15. Presence of non-participants | Was anyone else present besides the participants and researchers? | No |
| 16. Description of sample | What are the important characteristics of the sample? e.g. demographic data, date | See manuscript |
| *Data collection* |  |  |
| 17. Interview guide | Were questions, prompts, guides provided by the authors? Was it pilot tested? | See supplementary online material |
| 18. Repeat interviews | Were repeat interviews carried out? If yes, how many? | No |
| 19. Audio/visual recording | Did the research use audio or visual recording to collect the data? | All interviews were audio-recorded. |
| 20. Field notes | Were ﬁeld notes made during and/or after the interview or focus group? | Yes |
| 21. Duration | What was the duration of the interviews or focus group? | 40-120 minutes |
| 22. Data saturation | Was data saturation discussed?  *Yes. Despite small numbers, it was felt that this was reached.* | See column 2 |
| 23. Transcripts returned | Were transcripts returned to participants for comment and/or correction?  *No, although participants were given the option to discuss further and review if requested. Some participants chose to follow up, but only about the arts-based component of the study and these were all corresponded with or met in person, according to their preference, to follow up.* | See column 2 |
| **Domain 3: analysis and ﬁndings** |  |  |
| *Data analysis* |  |  |
| 24. Number of data coders | How many data coders coded the data?  *The interviewer coded the data, but coding was critically reviewed by the interviewer’s supervisees and a qualitative methods consultant.* | See column 2 |
| 25. Description of the coding tree | Did authors provide a description of the coding tree? | See manuscript |
| 26. Derivation of themes | Were themes identiﬁed in advance or derived from the data? | See manuscript |
| 27. Software | What software, if applicable, was used to manage the data? | See manuscript |
| 28. Participant checking | Did participants provide feedback on the ﬁndings?  *Participants did not provide feedback on the findings, but summaries of key findings will be made available to all once the PhD has been completed and sent out to those who had requested this.* | See column 2 |
| *Reporting* |  |  |
| 29. Quotations presented | Were participant quotations presented to illustrate the themes/ﬁndings? Was each quotation identiﬁed? e.g. participant number | See manuscript |
| 30. Data and ﬁndings consistent | Was there consistency between the data presented and the ﬁndings? | See manuscript |
| 31. Clarity of major themes | Were major themes clearly presented in the ﬁndings? | See manuscript |
| 32. Clarity of minor themes | Is there a description of diverse cases or discussion of minor themes? | See manuscript |
