# Supplementary material for: The Impact of Attempted Suicide on Young Adults: Learning from the Lived Experiences of UK Students in Further and Higher Education
Source: Healthcare (Basel). 2025 Dec 9;13(24):3222. doi: 10.3390/healthcare13243222 (PMC12733092; doi:10.3390/healthcare13243222)
Supplement: Supplementary file 1 [file healthcare-13-03222-s001.zip › healthcare-3941052-File S2.docx]

**Interview questions – Learning from Further and Higher Education students’ lived experiences of attempted suicide.**

**Before starting the interview**

- Thank the participant for agreeing to take part in the study and coming in today.
- Remind them about limits of confidentiality and ask if they have any questions about this or any part of the study.
- Explain that some of the questions may bring up difficult feelings and that they can take a break or stop at any point during the interview and that there is no obligation to continue, their safety and wellbeing is most important.
- If applicable, remind them that there is a Samaritans volunteer available if they want to take a break during the interview or after the interview.
- Explain that the interview is semi-structured and that I will therefore be reading some specific questions and may make notes during the interview.
- Remind the participant that the interview will be recorded and transcribed, but that data will be anonymised.
- Remind them that art making is part of the process and provide them with paper and materials to sketch whilst they speak, if they choose to.
- Check if they have any further questions.
- Ask if they are happy for the Dictaphone to be turned on at this point.

**Part 1 about you**

1. How are you finding college/university?
2. a. Please can you tell me about your experience of the transition between school and college/college and university (whichever applies)?

*(How did this make you feel? What was most challenging about the transition? Can you tell me more about your feelings? What, if any, support was available to you to help with the transition?)*

b. Is there anything that you think of which could help make transitions easier?  *(If so, what? Ask for specific examples, specific people at the college/university)*

1. What is the biggest challenge for you about college/university?

**Part 2 about the attempt and support**

As I mentioned earlier, I am going to ask you some more specific questions about your suicide attempt(s), experiences at college and/or university and your experience of any support structures at the institution. Please take your time to answer.

1. When did you first attempt suicide? *(if appropriate, although this study specifically relates to your experiences whilst at college/university, it would be helpful to know if you had thoughts/experiences before starting university.)*
2. How long did you feel suicidal before your (first) attempted suicide?
3. Did you seek any support when you first had suicidal thoughts? *(if yes, what support and how helpful/unhelpful was this, if no, why not)*
4. a. Has/is your suicide attempt/s impact(ing/ed) your college/university experience?
5. If yes, in what ways has it impacted/is it impacting your experience?
6. Have you told anyone about your attempt and if so, who have you told? *(refer back to the survey, friends, family members, support services, etc)*
7. a. What, if any support was available to you at the time – before and after?
8. Did you access any of this support? *(if appropriate refer to specific support they have identified on their initial survey)*
9. What was helpful?
10. What was unhelpful?
11. If no support was sought/accessed, why not?
12. What do you think could have been helpful at the time?
13. Is there anything you think could be helpful now?
14. Was there anybody in particular that was helpful? *(What was it about them/what did they do/say that was particularly helpful?)*
15. *(If not already evident from questions 5-8)*
16. Did/do your college/university know about the suicide attempts?
17. If yes, how has your college/university responded? *(How did this make you feel?)*
18. If not, why? *(what would encourage you to share this information?)*
19. What *(further)* measures in particular do you think the college/university could put in place?
20. What could they do that would make things worse?

**Part 3 lived experiences and impact on self**

1. a. When you first had suicidal thoughts whilst at college/university, what factors do you think contributed to these thoughts?

b. and your attempt?

1. How did you feel:
2. Immediately after
3. A few weeks later
4. Now
5. How has the attempt impacted on your:
6. Sense of self *(how you think about yourself, how you see yourself, how you feel about yourself, how you identify yourself)*
7. Sense of others *(how you experience other people, how you are in relationship with others – e.g. family, friends, (potential) partners, teachers, housemates, university staff in general (all the various roles), how you think others see you/how you think others would see you, your thoughts/feelings about others)*
8. Sense of society/the world *(your views of society/the world, your place in society/the world)*
9. What do you consider to be risk factors for yourself?
10. What has helped/helps you to keep going every day? Or What do you think has helped/helps you to be here today?

**Part 4 Reflections and challenging perceptions**

1. What would you like others to know about attempted suicide?
2. What message would you like to give to others? *(those who experience suicidal thoughts/behaviours, educators and the public)*
3. What is the biggest myth you would like to dispel/challenge?
4. Is there anything else you would like to share?
5. Any final thoughts? *(about attempted suicide, college/university, about taking part in the survey/the interview today or anything else)*

**Thank the participant for sharing their experiences.**
